# Am I My Brother’s Keeper? Moral Dimensions of Informal Caregiving in a Neoliberal Society

**DOI:** 10.1007/s10728-016-0313-7

**Published:** 2016-02-16

**Authors:** Ellen Meijer, Gert Schout, Tineke Abma

**Affiliations:** Department of Medical Humanities, VU University Medical Centre, De Boelelaan 1117, 1081 HZ Amsterdam, The Netherlands

**Keywords:** Moral ambivalence, Neoliberal policies, Informal care, Personal communities, Mental health

## Abstract

Within the current Dutch policy context the role of informal care is revalued. Formal care activities are reduced and family and friends are expected to fill this gap. Yet, there is little research on the moral ambivalences that informal care for loved ones who have severe and ongoing mental health problems entails, especially against the backdrop of neoliberal policies. Giving priority to one’s own life project or caring for a loved one with severe problems is not reconciled easily. Using a case study we illustrate the moral ambivalences that persons may experience when they try to shape their involvement and commitment when a relative is in need. The case comes from a research project which explores whether it is possible to reduce coercive measures in psychiatry by organizing a Family Group Conference. The purpose of the article is to explore what theoretical concepts such as ‘communities of fate’, ‘communities of choice’ and ‘personal communities’ add in understanding how persons shape their involvement and commitment when a family member experiences recurrent psychiatric crises.

## Introduction

In the past few decades there has been a debate about the role of the welfare state and the use of informal care or family care is revalued [28]. The role citizens themselves have in society is subject to change. They are summoned to be more independent from the welfare state and need to feel the responsibility to care for themselves and loved ones [33]. In Great Britain this development is known as ‘big society’; where the reformation of society and the transformation of the state to facilitate relationships between citizens are key elements [8]. In the Netherlands similar voices are heard, emphasizing the participation of citizens. Rather than depending on the government and having a ‘wait and see attitude’ they have to help others in need [46]. Trappenburg [46] describes how this requires different attitudes and actions from citizens and advocates for a change from passive to active solidarity. Furthermore, she explains, the moral call ‘help someone in need’, isn’t heard by everybody. This means that the burden of caring for someone in need is not always distributed in a fair way.

We recognize the difficulties in this transition where informal care becomes of more importance and want to add that persons may experience certain tensions between the willingness to care for a family member or neighbor and the time constraints that are inherent to living in a modern society; where being productive through labor is seen as important and where not everyone has the possibility to reduce their working hours [18, 45, 50]. How do persons shape their involvement with and commitment to others if there are conflicting expectations and moral dilemmas that accompany them? How do they want to relate to family, friends or peers? How is that reflected in forms of sacrifice and self-interest, of solidarity and calculation, of generosity and obligatory giving, of intimacy and aloofness?

These different feelings are well illustrated in the Italian/French film “Mia Madre” by Nanni Moretti where we see the struggles of a son and daughter in taking care of their ill mother [31]. The daughter, who pursues a career in film making experiences severe problems in dealing with her personal problems and professional career. Whereas the son takes leave from his job to care for his mother. Both struggle in this process and have different reasons for their actions. Giving priority to one’s own ambitions or to caring for loved ones seems to be surrounded by ambivalence. Different moral values may come into conflict with each other. Persons can also experience feelings of responsibility to support a loved one, based on love and generosity or on a sense of obligation towards others, or because one cannot bear the suffering of another.

The social and therapeutic value of social networks and social support can hardly be overestimated. The mental health of people can be enhanced and preserved when social support is present [35, 48, 49]. Persons with a mental illness also benefit from a committed network because with their help worsening of the situation can be prevented; the network has protective features [25, 43, 44]. Social support has a positive influence on coping with stress, self-control, a sense of optimism and hope [44, 47]. Having a social network that offers support or care, in short has a profitable effect on the mental health of people. But how self-evident is giving support or care to a family-member or friend with a mental illness? On the one hand solidarity, generosity and intimacy seem to be important values but simultaneously pursuing a career, earning money, and living your own life is seen as meaningful. Nowadays persons in Western societies have to deal with these moral ambivalences, because there are conflicting visions on what is considered good (a good life, good action, being a good family member).

How persons shape their involvement and commitment in the life of relatives in need has been studied extensively from a care ethics perspective by Lindemann. According to Lindemann [26] families can be seen as networks where love and trust are important (generally speaking). The affection in the family leads to a certain kind of vulnerability and induces responsibilities to care for and commit to each other [26]. However, Lindemann [26] also mentions that affection in the family can be combined with aspects like selfishness, indifference or carelessness. The moral ambivalences persons may experience when experiencing these different feelings when they care for someone, remain mostly unclear. The purpose of this article is to illustrate moral ambivalences in a neoliberal context, using both theory and the reality of a case, and to explore what theoretical concepts such as ‘communities of fate’, ‘communities of choice’ or ‘personal communities’ may add in understanding how people shape their involvement and commitment when a family member or friend experiences recurrent psychiatric crises.

We have carried out this study in the field of mental health care, a field where persons with severe and ongoing psychiatric problems and their families and friends often have complex relationships, where uncertainty and ambivalences to be involved in informal care occur [40]. These moral ambivalences also crop up in other areas of medical decision making. The length of the stay in hospitals and nursing homes is often related to the support of family members [52]. In all situations where compensation of self-care deficits is required, these dilemma’s arise.

The question we want to answer in this article is: How do persons shape their involvement with and commitment to a family member who has severe and ongoing mental health problems and how are they dealing with the moral ambivalences they experience in this situation? And what is the contribution of theoretical concepts from the field of sociology including ‘communities of choice’, ‘communities of fate’ and ‘personal communities’ in understanding these ambivalences? First we will discuss a case example that illustrates the moral ambivalences that family members and friends may encounter. Then we will point out theoretical concepts and will begin to explore what these concepts add in understanding how persons shape their involvement and commitment when a family member experiences recurrent psychiatric crises.

## Case Example

A case that illustrates the moral ambivalences family members may encounter comes from the research project ‘Family Group Conferencing in psychiatry.^1^’ In this project we investigated whether it is possible to reduce coercive measures in psychiatry by organizing a Family Group Conference (FGC). The psychiatric patients in this research project were at risk of being involuntary admitted in a psychiatric hospital, or sometimes still are admitted, due to risky or dangerous behavior in relation to their own safety or that of others. Research on the application of FGCs in Public Mental Health care shows that FGCs hold potential in preventing coercive measures in psychiatry [12]. This corresponds with the findings on the application of FGCs in youth care, where forced residential and foster care are prevented using FGCs [38, 51]. FGCs also reduce the risk of recidivism in juvenile crime [5, 9, 20] and are applied in elderly care as a means to reinforce relational autonomy and resilience [30].

FGCs originated in New Zealand and can be regarded as a decision-making model where the formal world of the government and organizations, comes together with the informal world of individuals, families and friends [14]. The conference is a meeting organized by the patient and FGC-coordinator, where plans are made along with family, friends and sometimes professionals to deal with the (problem) situation. An FGC gives the patient and social network the opportunity to deal with a (problematic) situation in a way that matches their own culture and lifestyle [12, 19, 30].

The potential of FGCs in avoiding coercive treatments in psychiatry lies in the widening of the circle of support, restoring of relationships and the evolvement of a knitted community that is available 24/7 [10, 11]. Plans can be made wherein social networks have a signaling function and can prevent escalation of an situation into a coercive treatment in collaboration with a client. The idea is that bringing these groups together offers possibilities for new solutions that improve the situation [7]. Furthermore FGCs appeal on the ownership and autonomy of the persons involved. This is contradictory to coercive measurements which most clients experience as a loss of ownership and infringing their fundamental rights [13, 22, 24].

In the research project 60 cases, where patients volunteered to participate in (the preparation of) an FGC, have been evaluated. The patients were included in three different mental health care organizations in the Netherlands. The methodology can be described as a responsive evaluation (see [1]). The purpose of the evaluation is to describe experiences during the process of the FGC, the character of the project is exploratory. Process and outcomes of the FGCs have been studied by interviewing the participants afterwards.

The case we selected has elements that are recognizable for persons in similar situations, holds learning potential and will be used to illustrate moral ambivalences persons experience in caring for a loved one. This is in line with Abma and Stake [3] who describe that case studies are especially fit to pinpoint the particularities and complexity of situations. Furthermore they specify that ‘if we are able to capture the essence and uniqueness of the case in all its particularity, it will reveal something that is universal’ [3, p. 1159]. By discussing the case, identifying patterns and combining it with theory we hope to achieve a better understanding of the complexity that comes along with caring for a relative in need in a meritocratic and neoliberal political context. We discuss a case in which a professional referred a mother of two daughters in their twenties, for an FGC. The mother has had a long history in psychiatry, characterized by strong mood swings caused by a bipolar disorder and behavior making it sometimes difficult for family and professionals to approach her.

Mother, her daughters and other family members have a history of incidents where the safety of all was at stake. The social network of the mother had shrunk to only a few family members. When the risk of being involuntary admitted to a psychiatric hospital was high and an FGC was being considered at the same time, she was staying in different places and avoided necessary forms of care. The professionals who were involved wanted to prevent an involuntary admission and suggested the possibility of an FGC. Mother didn’t want to participate in an FGC, her family, however, did and with them the dialogue for organizing an FGC continued. In the preparation of the FGC it became clear that the whole situation was a heavy burden for the family. The daughters have been through a lot with their mother and experienced psychological and emotional problems in their own lives. Despite everything the eldest daughter felt a strong responsibility towards caring for her mother. According to an aunt (sister of the mother) it was better if she would take less responsibility due to the experienced burden. This aunt (50) had also been through a lot with her sister and would rather walk away from the sorrow and misery. She had been insulted and harassed by her sister, yet she also indicated: ‘Of course, this is not what my sister wants herself’.

One of the sisters approached the aunt with the question whether she wanted to participate in the FGC. Actually she wanted to reject the request given the past. She had lost the faith in a positive outcome and doubted that the FGC would succeed. According to the aunt other attempts had failed too often. It required a lot from the aunt to participate in the FGC, also because she did not live in the same region and she had her own job and family. Furthermore it cost energy and time. It is imaginable that the aunt would experience feelings of doubt and guilt if she decided not to attend the conference. The daughters of her sister had already been through so much with their mother that they couldn’t do without her support. Despite the possible doubts and feelings of guilt that the aunt might experience, she had the choice not to interfere with the situation. This applied to everyone involved in the situation; all could choose to end the relationships and continue with their own lives.

The mother in this case might avoid contact with her family because of everything she put them through; the daughters probably realized that if they decided to walk away from their mother she had little reason to control her mood swings and seek for professional help. The aunt, in turn, would probably realize that her involvement in the situation could make a difference. The moral ambivalences in this case are far from unique. In cases of severe and ongoing mental health problems we see besides commitment all sorts of reservations, doubts, aloofness and hesitation.

## Neoliberal Identity

The willingness to practice a certain degree of commitment in the life of important others in need and the moral ambivalences experienced in this process are set against the backdrop of a neo-liberal discourse in which humans understand themselves as largely selfish individuals, making choices autonomously. Sandel [39] addresses the problem of the illusory promises of liberalism, that liberal subjects have autonomy and control over their lives. In such a context it seems like success is directly related to the persons own efforts, they have control over their own destiny and determine what happens in their lives. Harvey [16] describes the rise of neo-liberalism in recent decades, and mentions that individuals are personally responsible for their own success and failure. Markets and market oriented thinking expanded and market values reached into aspects of life that are traditionally driven by more nonmarket norms [39]. Success means that people can live up to the demands of society and when they fail they owe this to themselves. It isn’t hard to imagine that others may pose a threat in the quest of the individual to lead a successful life; this may create competition between individuals in society. Competition between individuals and also between organizations is an important virtue in neoliberalism [16]. Competition is believed to be the route to excellence and quality.

This fits the notion of negative liberty discussed by Berlin [6]. Negative liberty means that other persons should not interfere with your choices or hinder you from attaining a goal. Berlin [6] however mentions that persons are interdependent and that no one can ever act in such a way that he will never hinder the lives of others. He also states that some will need help, support or education before they can understand the concept of freedom and make use of it and asks the question ‘what is freedom to those who cannot make use of it? Without adequate conditions for the use of freedom, what is the value of freedom [6, p. 124]?’ Moreover, persons can be hindered by themselves to attain their goals and their freedom, they can experience internal barriers to achieve freedom. The help or support of others is necessary to achieve freedom and autonomy.

Fukuyama [15] discusses the ‘Hobbesian fallacy’, the idea that humans are primary individualistic beings and that they only enter society on the base of a rational calculation that social cooperation is the best option for them to achieve their own life goals and projects. Building on primate studies of chimpanzees he illustrates how our ape-like ancestors behaved in a social way and that chimpanzees and humans, have similar forms of social behavior. This makes it reasonable to assume that humans have always behaved in a social way and never were isolated individuals [15]. Nevertheless self-interest plays a role in relations, indeed it is important to recognize and accept the tension between selfishness, self-interest and social and altruistic behavior. If this succeeds the inconsistencies that people experience can be embraced and confined. As mentioned before, in current Western society the competitive side of people is stimulated. It remains hidden that we stand on the shoulders of our predecessors and cannot perform without the support of others. The myth of autonomy as self-determination continues. In this dominant meritocratic ideal success and failure are seen as the responsibility of individuals themselves [21]. Success is linked to personal excellence and failure to personal shortcoming [21]. Against this background, it appears to be acceptable when people ‘choose for themselves’; to a greater or lesser extent, everyone wants to be successful. In this market orientated thinking altruism, solidarity or generosity are resources that come under pressure when used. They are seen as scarce resources that need to be used carefully. Market tradition neglects the option, however, that our capacity to act altruistic or generously is increased with practice [39]. Sandel sees altruism and generosity as ‘muscles that develop and grow stronger with exercise’ [39, p. 130].

The foregoing is also relevant in understanding the moral ambivalences of the aunt; she is free to make the choice not to join her family and others could see this as an understandable choice. If she decides to join in the situation this means that she has to invest time, energy, attention and commitment and this is also required for her own family and job. Ultimately the aunt decides to engage in the situation, and is present at the conference. She says, ‘Maybe it’s a good thing to come together—with my nieces and the involved professional.’ She wants to be there for the daughters of her sister and she wants to make life more enjoyable for them. The decision of the aunt raises the question how it is possible that, in contexts where neoliberal values dominate, persons still show involvement in each other’s life and seem to care about relatives in need.

## The Moral Neoliberal?

In her book *The Moral Neoliberal* Muehlebach [32] discusses that despite the neoliberal context we live in, persons do actually behave morally and feel responsible for others. Although the established neoliberal order creates rational, utilitarian and instrumental acting persons, these persons do have an affective identity characterized by compassion and empathy. According to Muehlebach the market and the moral life have always coexisted. Besides the abundance of material wealth (in the west), there is also an abundance of so-called ‘virtues’; people are fanatically seeking success and wealth but also have a moral sense which makes them sympathize with others and care for them. Following Muehlebach compassion for others and ‘the coldness of wealth and success’ are not opposed to each other [32, p. 20].

Muehlebach’s [32] study of Italian volunteers in Milan, gives an image of a moral style of contemporary neoliberalism. She shows that volunteering is especially important in a relational way and is based on the affection of persons for each other. The government is withdrawing itself from the moral responsibility to care for citizens and shifts this responsibility to citizens themselves. The idea of ‘citizenship lived with the heart’ [32, p. 11] arises and this becomes the moral soil on which a community of solidarity can be reformed. The emergence of volunteering in Italy originates from a discourse in which a new kind of ethical commitment is seen between different groups in society. This commitment is based on a moral obligation rather than based on social rights and is not controlled or facilitated by the state [32]. In addition there is a need for meaning, commitment and love for persons in vulnerable situations. A volunteer in the study of Muehlebach describes this as “helping others while helping oneself” [32, p. 161]. Reciprocity between generations is not central, but a spirit of free gifting is [32].

Melucci [29] also points out that people can act in a selfless and altruistic way, and this can be seen as altruistic action. Altruistic behavior can be seen as a symbolic challenge, it is at odds with the rationality of calculating behavior and the efficiency of technology; because it is based on the commitment of people, their generosity and the desire for communication without a hidden program [29]. To give and offer without expecting a favor in return is an essential point rather than thinking in costs and benefits [2]. Altruistic action is established on a voluntary basis and has no direct (financial related) gain or benefit for those who act this way. People do get something in return, the opportunity to have an active and meaningful role in the life of important others and experience feelings of connection and belonging to a group. Major resources for altruistic action are gratitude and the ability to provide support or assistance [2].

The work of Muehlebach and Melucci makes it conceivable that expressions of solidarity and commitment do not need to be scarce and that there are resources outside the nuclear family. As Sandel mentions, altruism, solidarity and generosity have to be ‘exercised more strenuously to renew our public life’ [39, p. 130]. FGCs can play a role here, by utilizing the available resources and facilitating forms of solidarity.

## Communities

Even in neoliberal and meritocratic contexts supporting others or feeling compassionate for them is seen as an important value. It is however not hard to imagine that interfering in the life of someone with severe and ongoing mental health problems while dealing with the obligations of one’s own life, is challenging. A number of theoretical concepts are meaningful in understanding the shaping of involvement and commitment, and the moral ambivalences that persons experience doing so. First we will discuss these concepts and later on we will illustrate with the for mentioned case how useful these concepts actually are.

The first theoretical concept to be discussed is that of ‘communities of fate’. Stinchcombe [41] points out that ‘communities of fate’ can be seen as communities where the success and well-being of the individual is linked to that of the larger whole [41]. More explicit, according to Hirst [17], they can be seen as existential communities in which a person is born and then grows up in. ‘Communities of fate’ involve the sharing of a situation, process or particular fate. Despite the sharing of a fate, process or situation it is still possible for people to withdraw themselves from the care for others; to what extent can we speak of a shared fate persons have to submit to?

Hirst [17] mentions a shift from ‘communities of fate’ to ‘communities of choice’, which corresponds to the development of a neoliberal identity. The collective identities of people are increasingly influenced by their individual life projects. People build their lives according to their own preferences and want to be part of self-chosen, often temporary, communities [27]. This means that the family as traditional community is losing relevance. People want to be part of networks wherein others have the same preferences or interests or where they can meet contacts who would yield a career advantage. ‘Communities of choice’ emphasize the choice for a community, whereas ‘communities of fate’ imply a shared fate or destiny. Persons do not choose each other, they are connected through the situation, fate or process they share. Relations in chosen communities can be for example, friends, acquaintances or fellow members of an association or party. Whereas ‘communities of fate’ can be seen as family, sharing a fate and a blood tie. The distinction between ‘choice’ and ‘fate’ is however arbitrary; the family we have is given to us, but we can choose to keep distance. Likewise we can also choose to be involved with family, despite the shared fate. When living in a small town friends can be seen as part of a ‘community of fate’ because there are few options to choose your social contacts.

Pahl and Spencer [35] question the shift from ‘communities of fate’ to ‘communities of choice’. To demonstrate this shift demographic studies are used, which show an increasing number of divorces, greater social mobility, an increase in the number of highly educated people (especially women), an increase in the number of women in the labor market and the growth of non-heterosexual households [35]. This implies an increase in the importance of ‘communities of choice’. There are however other statistics showing that family ties are still relevant and there are few signs that friends are completely replacing family [34, 37]. It is more plausible to assume that traditional communities gradually disappear and new forms occur simultaneously [35, 42]. The vertical and more mandatory relationships that accompany traditional communities gradually merge with horizontal, chosen and more free forms of communities, where mandatory relationships play a smaller role [23]. Involvement with society and the communities where citizens are part of seem to have a different shape. Relationships with family or friends are not always clearly distinguishable. Persons have more flexibility and freedom organizing their personal relationships [4, 34]. For example in choosing how they want to live together and how they want to raise their children. Even though the fore mentioned freedom is limited by social and economic location a less standardized normative image of relations and a tendency for diversity is developing [4].

Taking account of the composition of relationships with family and friends Pahl and Spencer [35, 36] developed the concept of ‘personal communities’. With this they mean that persons have different relationships that vary in the degree of commitment and the extent to which they are given or chosen. The chosen relationships that Pahl and Spencer describe correspond to the previously described ‘communities of choice’. The given relationships resemble the ‘communities of fate’. Using the concept of ‘personal communities’ Pahl and Spencer [35, 36] study the social world of persons at the micro level and want to gain insight into the different relationships and communities where they are part of. Pahl and Spencer [35] recognize that different stakeholders, such as family and friends can play similar and contrasting roles. They also notice that a sharp distinction between given and chosen relationships is difficult to make and mention ‘a range of given and chosen relationships representing different forms and styles of suffusion’[35, p. 203].

Despite the neoliberal culture we live in people are willing to behave in altruistic or solidary ways. Within a FGC choices are made intersubjective, were values like altruism or generosity are negotiated upon with others in a community. Using the concept of personal communities brings insights who might be involved in a problematic situation. The concept makes social contacts outside ‘the given’ ones more distinguishable; a reservoir of social recourses opens up. This opens up opportunities to use FGCs as a means to alleviate the burden of the ‘community of fate’ and to expand the ‘given’ relationships with ‘chosen’ relationships.

## Returning to the Case

That the aunt in this case decides to interfere in the situation and doesn’t abandon her nieces because of what they’ve been through together can be described as a form of shared fate. In line with Pahl and Spencer [35, 36] the relationships in the case can be seen as given, they are based on the sharing of a situation. Sharing the difficult process as a mother, sister, daughter or aunt creates a bond that no one leaves behind easily. The involvement of the aunt seems to be primarily based on wanting to ‘ease the burden experienced by the daughters of her sister’ because they have been through a hard time. She apparently expects nothing in return.

Considering Muehlebach [32] and also Melucci [29] this can be seen as altruistic, because reciprocity seems less important and the aunt can play a meaningful role in the lives of her nieces. The expectation of reciprocity applies to a lesser extent because the mother and her daughters don’t have much to offer in return because of the struggles they experience in their own lives. During the process of the conference the family decides to start a procedure for a compulsory admission for their mother/sister because they can’t endure the situation any longer. In the following weeks however she stays at a psychiatric ward voluntary. The outcome of the conference revealed for the daughters and sister that they can actually support each other and also has created a better cooperation with the involved professional. Yet the situation remains fragile, durable support was not created. There is only a small group of persons involved, who seem to experience that they are bound together by a particular fate. The case illustrates how challenging it can be to shape informal care and offer durable support. Where the involvement first seems to be based on a shared fate later on the choice for professional help, with the appeal on an involuntary admission, is made. This illustrates that while on the one hand the relationships are experienced as given, they also have an aspect that is experienced as a choice; by reaching out to professional help. Just as mentioned by Pahl and Spencer [35, 36] a sharp or dichotomous distinction between given and chosen relationships seems difficult to make.

Looking back on the preparation of the conference there could have been more focus on the restoring of relationships and widening the circle of those involved. The small family network doesn’t feel capable to resolve the problems and keep supporting their mother or sister, considering that little reciprocity is to be expected. Specific for the mother in this situation it would probably be necessary to restore relationships because her social network has shrunk to only a few family members.

Building on the insights of Muehlebach [32], it would be fruitful to expand this small and fragile network with persons who are not connected by a shared fate. What would emerge when the small family group was expanded with neighbors, acquaintances or maybe volunteers or peers? An FGC could play an active role in restoring and/or strengthening the existing network, widening the circle and building a bridge between professionals and the social network. By widening the circle opportunities to include new points of view, considering the problematic situation, arise. In other words the ‘given’ relationships could be strengthened and ultimately widened with ‘chosen’ relationships.

## Conclusion and Discussion

In current Western societies persons experience moral ambivalences how to balance their own life style choices with caring for family members or friends who are in need. In a society with numerous options, there can be various ways to be involved in the life of others. How this is experienced or realized may differ. The concepts of ‘communities of fate’ and ‘communities of choice’ are used to explore how persons shape their involvement and commitment in the life of loved ones, and the moral ambivalence it brings about. The boundaries between the two communities are not dichotomous. To what extent do persons actually have a choice in relationships with friends or neighbors? Location (economic) and social, personal and material resources are of importance in experiencing the possibility of having choices and making them. Furthermore, there is also the possibility to evade obligations. Crucial is however the way in which persons experience their relationships, do they see them as given or chosen? Does the sharing of a particular fate play a role or are the relationships based on a thought-out choice?

The value of both concepts lies in identifying that persons can experience given and chosen relationships and that friends and family can play the same role. There’s more to it than just a connection through blood ties. The concept of ‘personal communities’ is useful in understanding the different relationships that persons have and in understanding their motives to be involved. We can however challenge the use of the word ‘personal’. It implies that relationships are centered around one person, and that this one person can decide who is part of that particular community. It is more plausible to assume that everyone in the community must contribute to maintain the relationship or community, fuelling reciprocity in the process. Lindemann [26] mentioned that carelessness or selfishness can also occur in families. Using a case we identified moral ambivalences persons may experience, and showed that making choices, about caring for a relative, is complex and full of tensions.

The three women in the case for example may indeed choose not to commit to the situation, but they decided otherwise because they experience a shared fate and have motives not to abandon or hurt their mother or nieces. The aunt shows how complex it can be to deal with different moral expectations, on the one hand she has the choice not to interfere with the situation and pursue self-interest but on the other hand she has feelings of generosity and a certain feeling of obligation towards her nieces to support them. The latter appears to be of more importance for the aunt than pursuing her self-interest and invest time in her own life only. Through discussing the case we identified feelings of self-interest, solidarity and calculation, generosity and obligatory giving, intimacy and aloofness. They seem to form a complex whole and exemplify the feelings and ambivalences that persons can encounter when caring for a relative in need.

The concept of personal communities makes insightful who are involved in a problematic situation, as described in the case. When a reservoir of social resources becomes visible, opportunities to widen the circle and strengthen relationships, with the help of a FGC, arise. Further research into the use of concepts like ‘personal communities’ and describing the different social networks involved and the motives and ambivalences they experience is necessary to comprehend what occurs in informal care against a meritocratic and neoliberal background.

Awareness for the moral ambivalences of persons who have both solidary aspirations and the desire to pursue their own life goals, provides opportunities for family, friends and professionals to consider how they want to shape their involvement and commitment. A positive tension between given and chosen relationships reveals itself. Both are of value, on the one hand we have our given relations with whom we share a fate but the choice to engage in meaningful relationships with others is also present. Despite the neoliberal and meritocratic culture people are relational beings and they need each other to form and give expression to relational autonomy. Muehlebach [32] argues that relations with others are not always based on reciprocity but can also be based on an a form of ‘charitas’ this means caring out of love or charity.

The social network in the example of the case, is small and worn out. The protective features of the network could be expanded through restoring relationships and widening the circle with volunteers or peers. Doing so this relational autonomy can be formed again. Moreover it may lead to a more balanced distribution of care and support and gives a hopeful perspective for those involved. The majority of psychiatric patients however experience social isolation or have difficulties to fuel the process of reciprocity, especially in those situations it seems necessary to widen the circle and alleviate the burden for the few persons that are involved and to strengthen those relationships. FGCs can be useful when persons need support or facilitation in restoring relationships or widening the circle by facilitating this process. Sandel [39] argues altruism needs to be evoked and practised, this requires changes in long term care structures and policies in the mental health arena. This also invokes a change from professionals in mental health care, policy makers and communities themselves. Further research into conditions for informal care and the role of communities and professionals herein is needed.

Am I my brother’s keeper? Individualisation and detraditionalization are transforming the way people respond to this call. The work of Muehlebach and the case example show, however, that there is hope for expanding social networks into a wider network of care, despite the time pressure of today’s life projects in a neoliberal context and holds a promise to widen the circle for those in need of support.

## Footnotes

1 The research proposal was approved by the Medical Ethic Review Committee of VU University Medical Centre. The Medical Research Involving Human Subjects Act (WMO) does not apply to the above mentioned study. An official approval of this study by the committee was not required (28th of March 2013).
